# Supplementary material for: Forward Modeling of Coronal Mass Ejection Flux Ropes in the Inner Heliosphere with 3DCORE
Source: Space Weather. 2018 Mar 7;16(3):216–29. doi: 10.1002/2017SW001735 (PMC5947730; doi:10.1002/2017SW001735)
Supplement: Supplementary file 1 — Supporting Information S1 [file SWE-16-216-s001.pdf]

Space Weather

Supporting Information for

Forward modeling of coronal mass ejection flux ropes in the inner heliosphere with 3DCORE

C. Möstl[1], T. Amerstorfer[1], E. Palmerio[2], A. Isavnin[2], C. J. Farrugia[3], C. Lowder[4], R.M. Winslow[3], J. M. Donnerer[1], E.K.J. Kilpua[2], P.D. Boakes[1]

[1]Space [1]Space Research Institute, Austrian Academy of Sciences, Graz, Austria.
[2]Department of Physics, University of Helsinki, Helsinki, Finland.
[3]Institute for the Study of Earth, Oceans, and Space, University of New Hampshire, Durham, NH, USA.
[4]Department of Mathematical Sciences, Durham University, Durham, United Kingdom.

Revision submitted to *Space Weather*, 2018 January 11

Contents of this file

Text S1
Table S1

Introduction

This is the mathematical description of the 3DCORE model, including a table that clarifies the relationships between flux rope handedness, the axial field direction and expected magnetic fields components in various coordinate systems.

Text S1.

The *3-Dimensional COronal Rope Ejection* or 3DCORE model

In order to model the situation that is taking place during the observation of coronal mass ejection flux ropes in interplanetary space, we have created the 3DCORE model, which is an acronym for *3-Dimensional Coronal Rope Ejection*.

Steps in 3DCORE

1. **The flux rope shape:** As the general shape of the ICME MFR shell, we use an elliptical torus that becomes narrower at the ends where the MFR is bending towards the Sun [similar to *Hidalgo et al.* 2012]. Note that for the magnetic field configuration in this paper we use a circular cross-section, thus we put forward here the general ellipse equations only for a simpler future improvement, so we set the aspect ratio a_r of the torus cross section to 1. We call this shape a *tapered torus*, which is described by the equations

(1)
$$
\begin{aligned}
& a = \rho_1(t), \\
& b = \rho_1(t) / a_r, \\
& c = 1, \\
& f_{aux} = \mathrm{c}\sin(\psi / 2), \\
& x = -(\rho_0(t) + a f_{aux} \cos(\phi)\cos(\psi), \\
& y = \rho_0(t) + a f_{aux} \cos(\phi)\sin(\psi), \\
& z = b f_{aux} \sin(\phi).
\end{aligned}
$$

Here, *ρ0(t)* is the major and *ρ1(t)* the minor radius of the torus, f_{aux} the auxiliary function which describes how the torus narrows towards the Sun [*Hidalgo et al.* 2012], *Ψ* are the coordinates along the torus axis and *Φ* the coordinate along its azimuthal direction, and *x,y,z* are the location of grid points of the CME in a cartesian coordinate system with the Sun in the origin, *x* positive towards the Earth, *y* towards solar west, and *z* normal to the solar equatorial *x-y* plane. These are *Heliocentric Earth Equatorial* (HEEQ) coordinates.

The torus center is at *ρ0(t)*. To describe the propagation of the torus in heliocentric distance, we use the center of the cross section at the torus apex, which is situated at *2ρ0(t)*. The torus axis thus forms a circle that always stays connected to the Sun.

The tapered torus shape can propagate in any direction, given by its HEEQ latitude *T* and longitude *P*. The torus is oriented at an angle *η*, the axis inclination angle, to the solar equatorial plane. The angle *η* ranges from 0° to 360°, and increases in a counterclockwise direction. The value of the inclination $\eta = 0°$ means that the axis is pointing to solar north, at 90° it is pointing eastward, at 180° towards solar south and at 270° toward west, with north and south directions corresponding to normals to the solar equatorial plane (HEEQ). The flux rope axis direction is thus unambiguously defined, ranging from 0 to 360 degrees. Rotating the shape according to three angles is done with the Euler-Rodrigues relations. First, we rotate

the torus by P degrees round the z axis, followed by a rotation of T around a modified y-axis, and finally by a rotation of the angle η around a modified x-axis. For the T and η rotations the y and x axes need to be changed according to spherical coordinates transformations, because after each applied rotation, the axis to which the rotation is applied changes.

2. **Adding the magnetic field:** We create a 3D MFR by placing 2.5D magnetic field cross sections along the MFR axis. Each cross section contains a grid of points, at steps in $d\Psi = 10°$, $d\Phi = 10°$ for the torus angles and dr= 0.01 AU in the radial direction in the torus cross section. In this first version of 3DCORE, we use circular-cross sections. However, due to the definition of the elliptical torus the model can be improved to include elliptical cross-sections in the future. Here, each of the circular torus cross-sections contains a 2.5D, uniform-twist Gold-Hoyle magnetic field [e.g. *Farrugia* et al. 1999, *Hu et al.* 2014], with B_ψ the axial component and B_Φ the azimuthal component, and a non-existent radial field component:

$$
\begin{aligned}
B_\phi &= \frac{H B_0(t)\tau r}{(1+\tau^2 r^2)}, \\
B_\psi &= \frac{B_0(t)}{(1+\tau^2 r^2)}, \\
B_r &= 0.
\end{aligned}
\qquad (2)
$$

The twist τ is the number of turns a field line makes per AU around the axus, r is the distance from the circle center, B_0 the axial field strength at the cross-section center, and H is sign of the handedness or chirality. The twist parameter is in the range of 1 to 20 [see *Hu et al.* 2014]. The handedness H and the inclination η make it possible to describe all 8 ICME MFR types [*Bothmer & Schwenn* 1998, *Mulligan et al.* 1998], which includes right- and left-handed ropes at various inclinations. Table 1 shows the combinations of η and H to produce a desired MFR type and the expected magnetic field components for a direct (apex) impact.

3. **Propagating the flux rope:** The propagation of the torus away from the Sun is described with the drag-based-model [DBM, *Vrsnak* et al. 2013] by

$$r_{apex}(t) = \pm\frac{1}{\gamma}\ln[1 \pm \gamma(V_0 - w)t] + wt + R_0,$$
$$V_{apex}(t) = \frac{V_0 - w}{1 \pm \gamma(V_0 - w)t} + w. \quad (3)$$

First, we define the apex position $r_{apex}(t)$ of the torus, which is the point with the furthest heliocentric distance. The movement of this point away from the Sun is described with a constant drag parameter γ (in the paper text we use $\Gamma = \gamma \times 10^7$ km^{-1}) and w as the background solar wind speed. $V_{apex}(t)$ is the apex speed as function of time, and V_0 the CME initial speed at distance R_0, determined from coronagraph observations.

The torus expands quasi-linearly [e.g. *Leitner et al.* 2007], with D_{1AU} being the MFR diameter at 1 AU, which is constrained by MESSENGER observations in such a way that the simulation magnetic field time series is consistent with the diameter at MESSENGER.

$$\rho_1(t) = \frac{D_{1AU}[r_{apex}(t)]^{1.14}}{2},$$
$$\rho_0(t) = \frac{r_{apex}(t) - \rho_1(t)}{2} \quad (4)$$

Equations (4) means that we first determine the torus minor radius *ρ1*, and then the position of the torus center at *ρ0*, which follows from the definition of the apex distance $r_{apex}(t)=2\rho0(t)+\rho1(t)$.

Th axial magnetic field decreases as a power law, with the exponent of -1.64, also taken from *Leitner et al.* [2007],

$$B_\rho(t) = B_{1AU}[2\rho_0(t)]^{-1.64}. \quad (5)$$

This equation (5) relates the magnetic field at 1 AU (B_{1AU}) to the axial magnetic field B_r at the current heliocentric distance of the torus axis, which is positioned at $2\rho_0(t)$.

4. **Making synthetic measurements:** A spacecraft can be placed at any position in the model heliosphere to extract synthetic in situ data along a given spacecraft position, given by latitude, longitude and heliocentric distance in HEEQ. As the CME expands and propagates into the heliosphere, at each timestep it is checked where the next grid point of the CME MFR is situated with respect to the spacecraft position. This needs to be defined because the spacecraft is of course never exactly on a position of a grid point in the CME MFR. To this end, the measurement parameter *m* is defined: for a synthetic measurement to be taken, $m < 0.05$ AU. Note that the choice of *m* is related to the grid resolution.

The last step of 3DCORE consists in the conversion of the MFR magnetic field components into the coordinate system of the spacecraft, which are often given in the RTN system (STEREO, MESSENGER), and for Earth the GSE and GSM systems are needed for comparison to magnetospheric indices. In the paper, however, we have used data converted to HEEQ and and SCEQ for easier comparison.

The unit vectors of the points in the torus are in cartesian coordinates given by:

$$\begin{aligned}
\vec{e}_r &= [\cos(\phi)\cos(\psi), \cos(\phi)\sin(\psi), \sin(\phi)] \\
\vec{e}_\phi &= [-\sin(\phi)\cos(\psi), -\sin(\phi)\sin(\psi), \cos(\phi)] \\
\vec{e}_\psi &= [-\sin(\psi), \cos(\psi), 0]
\end{aligned} \quad (6)$$

The magnetic field components in the torus coordinates and torus cartesian coordinates are given as (see equation 2):

$$\vec{B} = [B_r\vec{e}_r, B_\phi\vec{e}_\phi, B_\psi\vec{e}_\psi] = [B_x\vec{e}_x, B_y\vec{e}_y, B_z\vec{e}_z]. \quad (7)$$

The components *Bx*, *By* and *Bz* can be found by the dot product with the cartesian unit vectors

$$\begin{aligned}
B_x &= B_r\vec{e}_r \cdot \vec{e}_x + B_\phi\vec{e}_\phi \cdot \vec{e}_x + B_\psi\vec{e}_\psi \cdot \vec{e}_x \quad (8) \\
B_y &= B_r\vec{e}_r \cdot \vec{e}_y + B_\phi\vec{e}_\phi \cdot \vec{e}_y + B_\psi\vec{e}_\psi \cdot \vec{e}_y \\
B_z &= B_r\vec{e}_r \cdot \vec{e}_z + B_\phi\vec{e}_\phi \cdot \vec{e}_z + B_\psi\vec{e}_\psi \cdot \vec{e}_z
\end{aligned}$$

The results of these calculations are

$$
\begin{aligned}
B_x &= B_r\cos(\phi)\cos(\psi) - B_\phi\sin(\phi)\cos(\psi) - B_\psi\sin(\psi),\\
B_y &= B_r\cos(\phi)\sin(\psi) - B_\phi\sin(\phi)\sin(\psi) + B_\psi\cos(\psi),\\
B_z &= B_r\sin(\phi) + B_\phi\cos(\phi).
\end{aligned} \tag{9}
$$

Hence, we have the magnetic field components available in a cartesian system, but we have not yet rotated the cloud accordingly to its propagation direction and inclination. Thus, we now rotate this magnetic field vector (B_x, B_y, B_z) according to the Euler-Rodrigues relations, similar to point 2 above for the CME shape. Finally, these components need to be projected into coordinate system at a spacecraft to be comparable to real observations. For calculating the Dst index at Earth, the GSM system is used, which is done following the procedures in *Hapgood* [1992]. From HEEQ, a conversion to HAE and HEE is done. GSE is then produced by a sign reversal in HEE and the final GSM components are calculated from the GSE values.

The bulk plasma speed of the point that is measured inside the CME can be calculated from $V_{apex}(t)$ by a simple argument involving self-similarity: because the geometry of the CME shape does not change with time (only its size changes), the relations [*Möstl and Davies,* 2013] are valid:

$$
\begin{aligned}
&\frac{r_{apex}(t)}{V_{apex}(t)} = \frac{r_p(t)}{V_p(t)},\\
&V_p(t) = r_p(t)V_{apex}(t)/r_{apex}(t).
\end{aligned} \tag{10}
$$

Here, $r_p(t)$ and $V_p(t)$ are the heliocentric distance of the point where the magnetic field is measured.

Table S1. Relation of CME flux rope type to inclination angle η and handedness H in 3DCORE. Type: N = north, W = west, S = south, E = east. H: Left-handed = -1, right-handed = +1.

	SEN	SWN	NES	NWS	WNE	ESW	ENW	WSE
H	-1	1	1	-1	1	1	-1	-1
η	90	270	90	270	0	180	0	180
B (RTN, SCEQ)	$-B_N$ $-B_T$ $+B_N$	$-B_N$ $+B_T$ $+B_N$	$+B_N$ $-B_T$ $-B_N$	$+B_N$ $+B_T$ $-B_N$	$+B_T$ $+B_N$ $-B_T$	$-B_T$ $-B_N$ $+B_T$	$-B_T$ $+B_N$ $+B_T$	$+B_T$ $-B_N$ $-B_T$
B (GSE, GSM)	$-B_z$ $+B_y$ $+B_z$	$-B_z$ $-B_y$ $+B_z$	$+B_z$ $+B_y$ $-B_z$	$+B_z$ $-B_y$ $-B_z$	$-B_y$ $+B_z$ $+B_y$	$+B_y$ $-B_z$ $-B_y$	$+B_y$ $+B_z$ $-B_y$	$-B_y$ $-B_z$ $+B_y$
